# Asheville, 1903

**Published:** 1903-09

**Authors:** 


### Editorial.

### ASHEVILLE, 1903.

THE conventions that met at this justly celebrated mountain resort have closed their labors, and it becomes the editor's duty to sum up the results.

Before proceeding to consider their work it may be well to express a thought regarding the place of meeting. This "Land of the Sky," as it is lovingly called by those who know it best, is situated two thousand feet above sea level, and yet Asheville seems to be, and is, lower than any part of the surrounding country, and, in fact, while upon an elevated plateau, it is apparently in a valley surrounded by majestic mountains, range upon range coming into vision as the mists of cloud-land clear away from their summits. Other sections of our diversified landscape scenery in America may furnish—do furnish—mountains of greater altitude, but to those familiar with Rocky Mountain scenery there is nothing fairer or more inspiring than these elevations that surround and make Asheville what it is to-day,—the most beautiful health resort, not excepting Colorado, in the United States.

The conventions were called to meet at the "Battery Park" Hotel. This house is situated on what is politely called a hill, but to those whose lot was cast for a week in Asheville proper, and were forced to ascend this three times a day, the hill assumed the proportions of a mountain, and became a serious problem to those unused to mountain climbing. To those who were fortunate in securing comfortable rooms at the hotel the splendid view of this land of mountains amply repaid them for the inconveniences usual at convention hotels. Asheville, the town, is similar to most other towns,—full of active life and with a promise of growth into a beautiful city. At present it seems in a condition of transformation from the village to the city, containing much of both in general make-up. George W. Vanderbilt has been an inspiration to the people of Asheville. Through his wonderful transformation of his mountain estate into a dream of beauty he has brought a direct and positive influence upon the inhabitants and a pleasure to the artistic minds visiting it. With his millions expended he has made Asheville, and the latter has nobly responded.

The several conventions were well attended. The National Association of Dental Faculties had delegations from nearly its entire list of membership, which means from New England to California. The National Association of Dental Examiners was well attended. Both of these organizations met at the same time and in advance of the National Dental Association. The writer alludes, in another place, to one matter connected with the "Examiners." It is to be hoped that its further proceedings were more in consonance with dental progress than this, but the writer was not made familiar with its general work, and hence is unable to express any opinion as to its value.

The Association of Faculties confined its labor, as usual, to general legislation and to the discussion of proposed plans to advance dental educational methods. In a body of this kind individuality is a prominent trait, and this generally results in much friction, but this year there was less of this than usual.

The most prominent and, to many, unpleasant feature was the resignation of the Dental Department of Harvard University. This school claimed that, inasmuch as it proposed to increase its preliminary entrance standard upon all lines of work and in advance of any of the departments of universities in membership in the "Faculties," it was entitled to a continuance of the present three years' course. While it did not expect to reach the A.B. degree for some years, the aim of the school was ultimately to accomplish this as a preliminary requirement. With this high standard, present and prospective, Harvard claimed that it should not be required to extend its course to four years. This claim was given careful consideration in committee, but it was evident that to concede this meant a practical abandonment of the four years' course decided upon several years ago. This advance had only been secured through years of constant educational effort, and to accept Harvard's claim meant a retrograde to original conditions. Dental colleges have learned, through serious experience, that it is impossible to make a satisfactory dentist in three years, with the additional scientific requirements imposed in the present curriculum. The request of Harvard was, therefore, respectfully declined by vote in the main body, and thereupon this school presented its resignation.

The writer feels that this action of Harvard was not well considered. As a school it may possibly be able to stand alone, but is not something due the men who have struggled for years to make dental education in this country worthy the respect of university thought and traditions? It seems to the writer that when the better class of colleges have thus, amid untold difficulties, reached a commanding position, they should not have been requested by one of our greatest schools of learning to retrace their steps. That they were not willing to do this is to their credit, but, at the same time, there was a universal feeling of regret that an earnest and influential member of the organization should have felt compelled to withdraw at the moment the goal, long worked for, had been attained.

The dissatisfaction which has long been felt with many of the officials of the Faculties resulted in a revolution upon the election of officers. The Foreign Relations Committee was the centre of attack, but all suffered in common. All of the Foreign Relations Committee, with the exception of one, are new men. This committee has for a long period been a disturbing factor in the "Faculties" through its objectionable methods, and it is to be hoped that the new committee will profit by the lesson and not attempt the impossible.

The National Dental Association met at the time appointed, with Dr. Noel, of Nashville, in the chair. This gentleman presided with dignity, and his address was full of suggestions which, if carried out, should advance the character of the National Association. It is, however, evident to the thoughtful mind that our national body is degenerating, being infected with the commercialism of the age. The papers read were much in this direction. The amount of time expended on inlay filling was an inexcusable waste, as that subject has been threshed out beyond the possibility of extended interest. Lantern exhibits are proper, and within certain limitations have a value in teaching, but they are a bore of great magnitude in a dental convention. They necessarily require much time, and the man with his pointing rod is more intent describing his picture than he is in discussing the scientific side of the question he attempts to illustrate. The result is that those not particularly interested in this phase of dental work are barred out. There was a time in the history of the old "American" when certain parties monopolized entire evenings with matter mainly interesting to themselves. Some may remember the antagonisms that this action aroused and the final adoption of a resolution preventing this abuse in the future. We have it now in the National under another, but an equally objectionable, form.

What is needed is a more thorough systematizing of the work. Why should the members be forced to listen to three papers on inlay fillings? One certainly ought to cover the subject and permit a fruitful discussion from those directly interested. It would seem as though the plan of section meetings would be forced upon this body, unsatisfactory as this method is to many. The sections at present existing do not meet the requirements. At the present meeting there was very little time given for the exchange of views and less devoted to truly scientific subjects. This from a practical stand-point may not be a subject for regret, but all classes of mind need to be fed at such a gathering. The complaint was widely made that there was no result from the national body at all commensurate with the long distance travel and fatigue consequent upon that and the heat of the season. The writer has for years seen the same result, and it will continue until the faulty management is improved and a more propitious season for holding meetings is adopted. It is to be hoped that this will come in the very near future.

This article cannot be closed without an expression of gratification with the efforts of the local committee to make this meeting socially an enjoyable occasion. Nothing was spared on their part to make it one to be remembered, and that they succeeded in giving pleasure to many must be acknowledged with grateful thanks.

The conventions of 1903 have passed into history. It is our duty individually and collectively to now take up anew the work of preparing for the International Dental Congress of 1904, at St. Louis, and make it an assured success.

